# Familial Mediterranean fever with colonic lesions: A case report

**DOI:** 10.1002/deo2.246

**Published:** 2023-05-16

**Authors:** Hiroyuki Ariga, Reiko Kunisaki, Takeshi Ojima, Satoshi Suzuki, Kenta Okada, Junya Kashimura

**Affiliations:** ^1^ Department of Gastroenterology Mito Kyodo General Hospital Ibaraki Japan; ^2^ Inflammatory Bowel Disease Center Yokohama City University Medical Center Kanagawa Japan

**Keywords:** familial Mediterranean fever, prednisolone, colchicine, ulcerative colitis, 5‐aminosalicylate

## Abstract

A 26‐year‐old man with a history of ulcerative colitis treatment presented to our clinic with abdominal pain and fever. He had a history of bloody stools and abdominal pain at 19 years of age. A thorough examination by a medical practitioner, including lower gastrointestinal endoscopy, resulted in the diagnosis of ulcerative colitis. After induction of remission with prednisolone (PSL), the patient was treated with 5‐aminosalicylate. One year ago in September, his symptoms flared up again, and he was administered 30 mg/day of PSL until November of the same year. However, he was transferred to another hospital and referred to his previous doctor. During the follow‐up in December of the same year, flare‐ups of abdominal pain and diarrhea were reported. Upon review of the patient's medical history, familial Mediterranean fever was suspected because the patient had periodic fevers ≥38°C and symptoms that persisted even after oral steroid administration and were sometimes accompanied by joint pain. However, he was transferred again, and PSL was administered once more. The patient was referred to our hospital for further treatment. At the time of arrival, his symptoms did not improve with 40 mg/day of PSL, and endoscopy and computed tomography revealed thickening of the colon, with no abnormality in the small intestine. Suspecting familial Mediterranean fever‐associated enteritis, the patient was administered colchicine, resulting in an improvement in symptoms. Furthermore, an examination of the MEFV gene showed a mutation in Exon5 (S503C), and atypical familial Mediterranean fever was diagnosed. Endoscopy after colchicine treatment revealed that the ulcers improved remarkably.

## INTRODUCTION

Familial Mediterranean fever (FMF) is an autoimmune disease caused by mutations in the *MEFV* gene and is characterized by periodic fever associated with peritonitis, pleuritis, and arthritis. Prevalent mutation sites in the *MEFV* gene are known to vary by race, and approximately 500 patients with FMF have been reported in Japan.^1^ Gastrointestinal mucosal damage in FMF is rare; however, gastrointestinal lesions resembling inflammatory bowel disease have been reported. In patients with atypical inflammatory bowel disease, periodic fever, and joint symptoms, FMF should be considered for differential diagnosis.

## CASE REPORT

**FIGURE 1:**
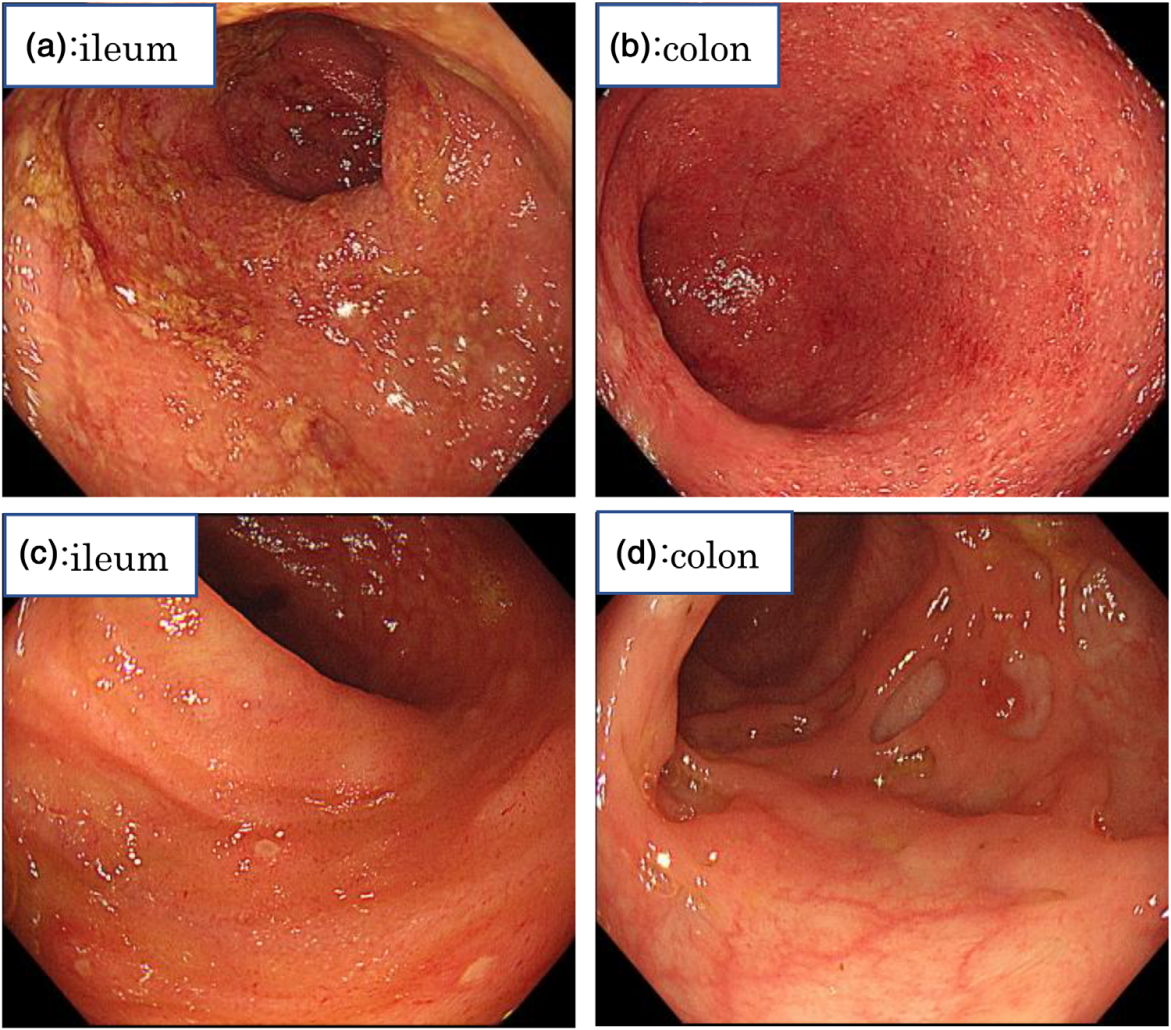
(a, b) Colonoscopy revealed loss or reduction of vascular permeability throughout the colon mucosa, with coarse mucosa, erythema, and adhesions of purulent secretions. (c, d) After treatment with colchicine, the symptoms improved, and endoscopy revealed multiple villous lesions in the small intestine and improvement in the erythema and rough mucosa previously observed throughout the colon.

**FIGURE 2:**
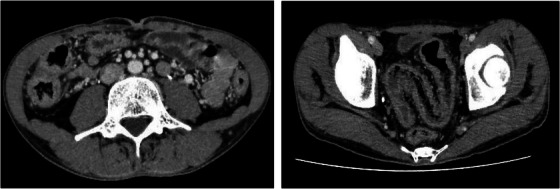
Contrast‐enhanced computed tomography revealed thickening of the colon and rectal wall and no small bowel lesions or vascular abnormalities.

A 26‐year‐old man presented to our clinic with abdominal pain and fever. He had a history of ulcerative colitis treatment, bloody stools, and abdominal pain at 19 years of age. He had visited a medical practitioner who performed a thorough examination, including a colonoscopic fiberscope and was diagnosed with ulcerative colitis. The patient was treated with 5‐aminosalicylate (5ASA) after induction of remission using prednisolone (PSL). One year ago in September, his symptoms flared up again, and he was administered 30 mg/day of PSL until November (Table 1). He moved to a new location and was referred to his previous doctor who referred him to our hospital. During his follow‐up in December of the same year, he complained of flare‐ups of abdominal pain and diarrhea. Blood tests revealed a white blood count of 8400/μl (87.5% neutrophils), a C‐reactive protein level of 24.78 mg/dl, and elevated amyloid protein (1568.5 μg/ml). Colonoscopy revealed loss or reduction of vascular permeability throughout the mucosa of the colon, with coarse mucosa, erythema, and adhesions of purulent secretions (Figure 1a,b). Therefore, 30 mg/day of PSL was administered as treatment. Upon review of the patient's medical history, FMF was suspected because the patient had periodic fevers of 38°C or higher and symptoms that persisted even after oral steroid administration and were sometimes accompanied by joint pain. Although familial Mediterranean fever was suspected based on the course of the disease, the patient was referred to our hospital because he had moved. At the time of presentation, his symptoms did not improve even with the administration of 40 mg/day of PSL, and computed tomography revealed thickening of the colon and no abnormality in the small intestine (Figure 2). This was clinically a typical case of FMF based on the Tel‐Hashomer criteria, but not according to Japanese guidelines. After the administration of colchicine and explanation to the patient, the MEFV gene was examined and was associated with a heterozygous mutation in Exon5 (S503C). Based on the above, the patient was diagnosed with atypical FMF according to the Japanese guidelines. After treatment with colchicine, the symptoms improved, and endoscopy showed multiple villous lesions in the small intestine and improvement in the erythema and rough mucosa previously observed throughout the colon (Figure 1c,d). The patient remained in remission (Table 1).

**TABLE 1:** Patient's clinical course

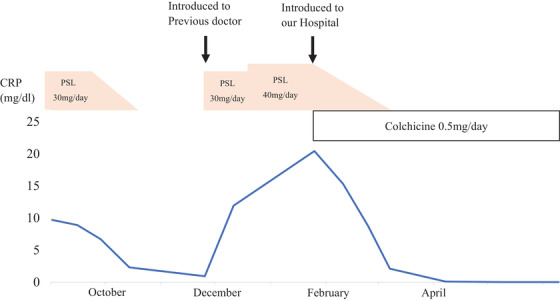

## DISCUSSION

FMF, an autoinflammatory disease, is characterized by periodic fevers with pain in the abdomen and chest due to serositis.^2^ More than 100,000 cases of FMF have been reported globally, and the prevalence (one in 200 individuals) of FMF is higher in the Mediterranean region.^3^

In 1997, the familial Mediterranean fever gene (*MEFV* gene) located on the short arm of chromosome 16 was identified to be responsible for FMF.^4^ The MEFV gene is located on chromosome 16 and consists of 10 exons.^4^ Thus far, >50 mutations have been reported globally, and >74% of mutations have been identified in one of the five exons, Exon10 (M694V, V726A, M694I, and M680I) and Exon2 (E148Q).

Recently, several cases of FMF have further been reported in Japan. In other countries, FMF onset usually occurs in childhood; however, in Japan, patients aged >20 years account for nearly 40% of cases. Moreover, the incidence of adult FMF in Japan is higher than that in other countries. The most common symptom of FMF is fever (95.5%), followed by abdominal pain (62.7%), chest pain (35.8%), joint pain (31.3%), and skin rash (7.5%). The incidence of abdominal pain as a symptom in Japan is lower than that in other countries. FMF is diagnosed based on the Tel‐Hashomer criteria.^5^ According to these criteria, FMF is classified into two types (complete or incomplete), depending on the severity of symptoms and the grade and periodicity of the fever.

The main pathology of FMF is serosal inflammation; however, patients may also present with severe abdominal pain associated with peritoneal irritation. Owing to the similarity of symptoms between FMF and Crohn's disease, some patients with FMF have been misdiagnosed and treated for inflammatory bowel disease. These are often not identified in cases with concomitant intestinal lesions.

*MEFV* gene expression has been reported to increase in the inflamed bowel mucosa of patients with inflammatory bowel disease, suggesting that FMF may be associated with mucosal lesions as well as serosal lesions.^6^ Some cases of FMF associated with colonic lesions do not present with genetic abnormalities of Exon10 (M694V, V726A, M694I, and M680I) or Exon2 (E148Q; Table 2), making the diagnosis of FMF even more difficult.

**TABLE 2:** Case report of familial Mediterranean fever with intestinal and/or colon lesions in Japan

	**Sex**	**Age** **(years)**	**Location**	**Endoscopy findings**	**MEFV** **gene mutation**	**Treatment**	**Author**	**Journal**	**Year**
1	F	25	Terminal ileum	Ulcer	E148Q	Colchicine, Infliximab: not effective Etanercept: effective	Kasamaki et al.	*Intern Med*	2019
2	F	42	Right‐sided colon	Decreased vascular permeability, erythema, erosion, and pseudo polyposis	G304R	Colchicine	Arasawa et al.	*Lancet*	2012
3	M	30	Jejunum	Mucosal edema	M694I	Colchicine	Matsumotoet al.	*BMC Res Notes*	2014
4	M	20s	Colon	Decreased permeability, erythema, edema, and granular mucosa	Silent mutation	Colchicine	Yamamoto et al.	*Stomach and Intestine*	2015
5	F	57	Jejunum	Erosion and mucosal edema	None	Colchicine	Sato et al.	*Gastroenterol Endosc*	2015
6	F	28	Colon (cecum)	Erosion	E148Q and M694I	Colchicine	Hidaka et al.	*Gastroenterol Endosc*	2015
7	M	66	Jejunum and ileum	Decreased permeability and erythema	E84K and P369S	Colchicine, infliximab	Kitade et al.	*Intern Med*	2015
8	F	68	Colon (cecum)	Decreased permeability, erythema, and edema	G304R	Colchicine	Torisu et al.	*Gastroenterology*	2017
9	F	38	Colon (cecum)	Ulcer	E148Q and M694I	Colchicine	Sato et al.	*Gastroenterol Endosc*	2018
10	F	30	Jejunum	Mucosal edema	G148G and M694I	Colchicine	Kawasaki et al.	*J Jpn Surg Assoc*	2019
11	M	37	Jejunum, ileum	No endoscopy Stenosis on fluoroscopy	Exon3 and exon8	Colchicine	Kasamaki et al.	*Intern Med*	2019
12	F	70	Ascending colon and ileum	Erosion and mucosal edema	G304R	Colchicine	Tanaka et al.	*Stomach and Intestine*	2019
13	F	80s	Colon	Erosion, mucosal edema, and ulcer	R202Q	Colchicine	Iida et al.	*IBD Res*	2019
14	M	20s	Ileum and colon	Erosion and mucosal edema	S503C	Colchicine: not effective Canakinumab: treatment	Iida et al.	*IBD Res*	2019
15	M	23	Ileum and colon	Decreased permeability and edema	S503C	Canakinumab	Yokoyama et al.	*CJG*	2021
16	M	26	Ileum and colon	Decreased permeability, erythema, edema, and granular mucosa	S503C	Colchicine	Our case	‐	‐

M, male; F, female; MEFV, Mediterranean fever.

In atypical FMF, mutations in the MEFV gene Exon10 are rare and often accompanied by mutations in Exon1 (E84K), Exon2 (E148Q, L110P‐E148Q, R202Q, and G304R), and Exon3 (P369S‐R408Q) and Exon5 (S503C) mutations.^7^ MEFV mutations in atypical FMF tend to have few Exon10 mutations and many heterozygous mutations,^7^ which was consistent with our case. Small bowel stenosis has already been reported in patients with FMF outside Japan. When stenosis is caused by FMF, peritonitis and an increase in intraperitoneal exudate led to the development of intestinal adhesions. In FMF patients with gastrointestinal lesions, typical cases account for approximately 30% and atypical cases account for approximately 70% of the cases.^7^ The part of the gastrointestinal tract affected by FMF often includes the large intestine and jejunum; however, the entire gastrointestinal tract can also be affected. Characteristic findings in lower gastrointestinal lesions include UC‐like circumferential mucosal findings, observed in more than half of cases, but rectal lesions are often absent; pseudo polyposis; and the presence of longitudinal ulcerative lesions and stenosis similar to those in patients with Crohn's disease.^8^ Some cases of FMF have been treated with colchicine alone, but there are also reports of cases treated with biologics (Table 1).

We experienced a case of FMF with colorectal lesions that were difficult to diagnose. The patient had periodic fever and arthritis, and the MEFV gene test showed Exon5 (S503C), which was suspected to be FMF and a diagnosis of atypical FMF was diagnosed. FMF may be present in patients diagnosed with unclassified inflammatory bowel disease and should be considered in their diagnosis.

## CONFLICT OF INTEREST STATEMENT

Dr Kunisaki had previously received funded collaborative research grants from Takeda, AbbVie, Tanabe Mitsubishi, Janssen, Eli Lilly, Novartis, and Viatris. Dr Kunisaki had previously received consulting fees from Zeria and speaker honorarium from Sandoz, AbbVie, EA pharma, KYORIN, JIMRO, Takeda, Tanabe Mitsubishi, Nippon Kayaku, Janssen, and Pfizer. Dr Kunisaki participated in a conference as an expert testimony for Nippon Kayaku.

## References

[1] Ogita C , Matsui K , Kisida D *et al*. A retrospective analysis of 7 cases of familial Mediterranean fever. Jpn J Clin Immunol 2017; 40: 21–7. 10.2177/jsci.40.21 28539550

[2] Alghamdi M . Familial Mediterranean fever, review of the literature. Clin Reumatol 2017; 36: 1707–13. 10.1007/s10067-017-3715-5 28624931

[3] Ben‐Chetrit E , Touitou I . Familial Mediterranean fever in the world. Arthritis Rheum 2009; 61: 1447–53. 19790133 10.1002/art.24458

[4] French FMF Consortium . A candidate gene for familial Mediterranean fever. Nat Genet 1997; 17: 25–31. 9288094 10.1038/ng0997-25

[5] Livneh A , Langevitz P , Zemer D *et al*. Criteria for the diagnosis of familial Mediterranean fever. Arthritis Rheum 1997; 40: 1879–85. 9336425 10.1002/art.1780401023

[6] Villani AC , Lemire M , Louis E *et al*. Genetic variation in the familial Mediterranean fever gene (MEFV) and risk for Crohn's disease and ulcerative colitis. PLoS One 2009; 4: e7154. 19784369 10.1371/journal.pone.0007154 PMC2745755

[7] Nakase H . The endoscopic diagnosis of familial Mediterranean fever gene‐related enterocolitis. Endoscopia Digestiva 2020; 32: 286–9.

[8] Nakase H . The characteristics of intestinal lesions of familial Mediterranean fever gene‐related enterocolitis. Gastroenterol Endosc 2019; 61: 2455–65.

